# Clinical Recommendation for the Use of Injectable Lenacapavir as HIV Preexposure Prophylaxis — United States, 2025

**DOI:** 10.15585/mmwr.mm7435a1

**Published:** 2025-09-18

**Authors:** Rupa R. Patel, Karen W. Hoover, Allison Lale, Janet Cabrales, Katrina M. Byrd, Athena P. Kourtis

**Affiliations:** 1 Division of HIV Prevention, National Center for HIV, Viral Hepatitis, STD, and TB Prevention, CDC.

**Summary**

**What is already known about this topic?**

HIV preexposure prophylaxis (PrEP) reduces HIV incidence; however, adherence to available PrEP regimens is suboptimal. In June 2025, FDA approved injectable lenacapavir (LEN) administered every 6 months as HIV PrEP, based on results from two randomized controlled trials (RCTs) that reported LEN efficacy at reducing HIV infection as 100% among females and 96% among a primarily male trial population over a follow-up of 52 weeks.

**What is added by this report?**

Based on efficacy and safety demonstrated by the two RCTs, the CDC PrEP Guidelines Work Group strongly recommends lenacapavir injections as an HIV PrEP option in persons weighing ≥77 lbs (≥35 kg) who would benefit from PrEP.

**What are the implications for public health practice?**

LEN has the potential to improve PrEP adherence and thus enhance HIV prevention.

## Abstract

In 2023, approximately 39,000 persons received a diagnosis of HIV in the United States. Although HIV preexposure prophylaxis (PrEP) is highly effective in preventing HIV infection, acceptance of, adherence to, and persistence taking the available oral and injectable PrEP regimens have been suboptimal. CDC PrEP guidelines published in 2021 include two oral tenofovir-based regimens and cabotegravir, the only injectable PrEP regimen approved by the Food and Drug Administration (FDA) at that time. In June 2025, FDA approved injectable lenacapavir (LEN), administered every 6 months, as HIV PrEP based on results from two randomized controlled trials (PURPOSE 1 and PURPOSE 2). The CDC PrEP Guidelines Work Group assessed evidence for the efficacy and safety of LEN PrEP using the Grading of Recommendations, Assessment, Development and Evaluation (GRADE) approach. The two trials reported LEN efficacy at reducing HIV infection as 100% among females and 96% among a primarily male trial population, compared with the estimated background HIV incidence (or no use of PrEP) over a follow-up of 52 weeks. No significant safety concerns were identified in the trials. The most common adverse events were mild (grade 1) to moderate (grade 2) injection site reactions. Based on a high certainty of evidence for the efficacy and safety of LEN as assessed by the GRADE analysis, subcutaneous injection of LEN every 6 months is strongly recommended as a PrEP option in persons weighing ≥77 lbs (≥35 kg) who would benefit from PrEP. LEN has the potential to improve PrEP adherence and thus enhance HIV prevention in the United States.

## Introduction

In 2023, approximately 39,000 persons received a diagnosis of HIV in the United States (*1*). HIV preexposure prophylaxis (PrEP) is highly effective at preventing HIV infection (*2*,*3*); however, implementation has been challenged by low acceptance of, adherence to, and persistence taking currently available PrEP options in the United States: 1) daily oral tenofovir-based regimens and 2) injectable cabotegravir administered every 2 months (*4*–*8*). Multiple national assessments have found that approximately one half of oral daily PrEP users discontinue it within 6–12 months of beginning prophylaxis (*4*–*6*). On June 18, 2025, the Food and Drug Administration (FDA) approved injectable lenacapavir (LEN) for PrEP, an HIV-1 capsid inhibitor administered every 6 months (*9*).

This report presents the CDC 2024–2025 PrEP Guidelines Work Group’s assessment of evidence from two randomized controlled trials (*10*,*11*) of the efficacy and safety of injectable LEN, administered every 6 months subcutaneously, for HIV PrEP, and provides a clinical recommendation for its use. The recommendation in this policy note updates the 2021 CDC PrEP Clinical Practice Guideline (*12*) by including LEN as an option for PrEP.

## Methods

### Guideline Development Process

This recommendation was drafted according to CDC’s guideline development standards (*13*). The work group developed the LEN PrEP recommendations as an update to the 2021 CDC PrEP Clinical Practice Guideline (*12*) using the Grading of Recommendations, Assessment, Development and Evaluation (GRADE) approach (*14*). The CDC 2024–2025 PrEP Guidelines Work Group comprised members from CDC’s Division of HIV Prevention who convened and met regularly during May 2024–January 2025. Members were selected for their experience (including clinical practice or research), expertise in PrEP-related topics, or experience in CDC guideline development. The work group generated a research question, conducted a systematic literature review, and synthesized evidence to guide work group discussions (*14*–*16*).

The focused research question for evidence synthesis was, “Should injectable LEN be included as an option for persons who would benefit from HIV PrEP?” The population, intervention, comparison, and outcomes (PICO) framework was used, and the components were as follows: adults and adolescents weighing ≥77 lbs (≥35 kg) (weight criterion per LEN FDA label indication) who would benefit from PrEP for HIV (the population); LEN PrEP (the intervention); no or daily oral tenofovir disoproxil fumarate-emtricitabine (TDF/FTC) PrEP (the comparison groups); and PrEP efficacy (HIV incidence rate ratio) compared with no PrEP use or daily oral TDF/FTC use, adverse events (excluding injection site reactions), injection site reactions, and grade 3–5 adverse events (severe, life-threatening, or fatal adverse events) (the outcomes). LEN compared with injectable cabotegravir PrEP was not evaluated in the absence of randomized evidence comparing the two. All outcomes were deemed critical by the CDC 2024–2025 PrEP Guidelines Work Group to develop the LEN PrEP recommendations. The work group also considered acceptability, feasibility, resource use, and harms and benefits while developing the recommendations. This activity was reviewed by CDC, deemed not research, and conducted consistent with applicable federal law and CDC policy.*

### Systematic Literature Review

The work group conducted a systematic literature search using Medline, Embase, Cochrane, CINAHL, Scopus, and Clinicaltrials.gov databases to identify published, randomized controlled trials for the population and intervention from database inception through February 21, 2025. Two reviewers independently screened 62 titles and abstracts, excluding 60 records because they were 1) in a non-English language, 2) conference abstracts, 3) trial protocol listings, 4) HIV treatment studies, 5) pharmacokinetic trials, or 6) animal and molecular studies. Two full-text reports were reviewed and included for evidence synthesis; the GRADE assessments were presented to the work group in February 2025 (Table 1) (Table 2). Recommendations were made by work group consensus (at least two thirds of votes). Work group members, none of whom reported conflicts of interest, reviewed the draft guidelines. The work group then created an external peer review plan, and the reviewers’ comments were incorporated into the final document.

**TABLE 1:** Efficacy evidence for injectable lenacapavir for HIV preexposure prophylaxis compared with no preexposure prophylaxis and daily oral preexposure prophylaxis — Grading of Recommendations, Assessment, Development and Evaluation, United States, 2025

Factors	HIV infection comparison groups	
	Lenacapavir vs. background HIV incidence (no PrEP)	Lenacapavir vs. TDF/FTC
**Certainty assessment**		
No. of studies	Two*	Two*
Study design	Background incidence study design^†^	Randomized trials
Risk of bias^§^	Serious	Not serious
Inconsistency	Not serious	Not serious
Indirectness	Not serious	Not serious
Imprecision	Not serious	Not serious
Other considerations	Very strong association^¶^	None
**No. of HIV infections/No. of participants (mITT analysis)**		
Injectable lenacapavir	**PURPOSE 1** 0/2,134	**PURPOSE 1** 0/2,134
	**PURPOSE 2** 2/2,179	**PURPOSE 2** 2/2,179
Comparator	**PURPOSE 1** 92/8,094	**PURPOSE 1** 16/1,068
	**PURPOSE 2** 45/4,634	**PURPOSE 2** 9/1,086
**Effect estimate**		
Incidence rate ratio (95% CI)	**PURPOSE 1** 0 (0–0.04)	**PURPOSE 1** 0 (0–0.10)
	**PURPOSE 2** 0.04 (0.01–0.18)	**PURPOSE 2** 0.11 (0.02–0.51)
Certainty	High	High
Importance	Critical	Critical

**Abbreviations:** mITT = modified intention to treat; PrEP = preexposure prophylaxis; TDF/FTC = tenofovir disoproxil fumarate-emtricitabine.

* Bekker LG, Das M, Abdool Karim Q, et al; PURPOSE 1 Study Team. Twice-yearly lenacapavir or daily F/TAF for HIV prevention in cisgender women. N Engl J Med 2024;391:1179–92. https://doi.org/10.1056/NEJMoa2407001; Kelley CF, Acevedo-Quinones M, Agwu AL, et al; PURPOSE 2 Study Team. Twice-yearly lenacapavir for HIV prevention in men and gender-diverse persons. N Engl J Med 2025;392:1261–76. https://doi.org/10.1056/NEJMoa2411858.

^†^ The comparison was based on a background incidence study design; the study design was nonrandomized.

^§^ The RiskofBias2tool was used to assess the risk for bias in both trials for the comparator of TDF/FTC (randomized trials) and the ROBINS-Itool (Risk of Bias in Non-Randomized Studies of Interventions) for the comparator of background HIV incidence (nonrandomized trials).

^¶^ Association (threshold of rate ratio <0.20) observed between the intervention and the outcome when compared with the comparator group.

**TABLE 2:** Adverse events from injectable lenacapavir for HIV preexposure prophylaxis compared with daily oral preexposure prophylaxis — Grading of Recommendations, Assessment, Development and Evaluation, United States, 2025

Factors	Adverse events		
	Any grade (excluding injection site reactions)	Grade 3, 4, or 5* (including injection site reactions)	Injection site reaction (any grade)
**Certainty assessment**			
No. of studies	Two^†^	Two^†^	Two^†^
Study design	Randomized trials	Randomized trials	Randomized trials
Risk of bias	Not serious	Not serious	Not serious
Inconsistency	Not serious	Not serious	Not serious
Indirectness	Not serious	Not serious	Not serious
Imprecision	Not serious	Not serious	Not serious
Other considerations	None	None	None
**No. of adverse events/No. of participants (%)**			
Injectable lenacapavir	**PURPOSE 1** 1,631/2,138 (76.3)	**PURPOSE 1** 92/2,138 (4.3)	**PURPOSE 1** 1,470/2,138 (68.8)
	**PURPOSE 2** 1,607/2,183 (73.6)	**PURPOSE 2** 104/2,183 (4.8)	**PURPOSE 2** 1,816/2,183 (83.2)
Oral daily TDF/FTC	**PURPOSE 1** 830/1,070 (77.6)	**PURPOSE 1** 52/1,070 (4.9)	**PURPOSE 1** 363/1,070 (33.9)
	**PURPOSE 2** 803/1,088 (73.8)	**PURPOSE 2** 66/1,088 (6.1)	**PURPOSE 2** 756/1,088 (69.5)
Certainty	High	High	High
Importance	Critical	Critical	Critical

**Abbreviation:** TDF/FTC = tenofovir disoproxil fumarate-emtricitabine.

* Severe (grade 3), life-threatening (grade 4), or fatal (grade 5) adverse events.

^†^ Bekker LG, Das M, Abdool Karim Q, et al; PURPOSE 1 Study Team. Twice-yearly lenacapavir or daily F/TAF for HIV prevention in cisgender women. N Engl J Med 2024;391:1179–92. https://doi.org/10.1056/NEJMoa2407001; Kelley CF, Acevedo-Quinones M, Agwu AL, et al; PURPOSE 2 Study Team. Twice-yearly lenacapavir for HIV prevention in men and gender-diverse persons. N Engl J Med 2025;392:1261–76. https://doi.org/10.1056/NEJMoa2411858.

## Rationale and Evidence

### Clinical Efficacy Trials for LEN as HIV PrEP 

The evidence base comprised two phase-3 multisite, double-blinded, randomized controlled clinical efficacy trials, PURPOSE 1 and PURPOSE 2, which were conducted during June 2021–September 2024 (*10*,*11*). The PURPOSE 1 trial enrolled females aged 16–25 years in South Africa and Uganda.^†^ This trial compared HIV incidence among those who received injectable LEN every 6 months (using an initial 2 days of oral loading doses) with the estimated background HIV incidence in the screened population (no PrEP use; primary endpoint) and HIV incidence among participants who received daily oral TDF/FTC (secondary endpoint) (*10*). The PURPOSE 1 trial also assessed the efficacy of daily oral tenofovir alafenamide-emtricitabine (TAF/FTC), a medication that has not been approved by FDA as PrEP for women, compared with no PrEP use and daily oral TDF/FTC use, and assessed safety outcomes for LEN compared with both tenofovir-based options (*10*). 

The PURPOSE 2 trial primarily enrolled males (see Table 1 in the trial report [*11*]) aged 16–80 years in 92 trial sites in Argentina, Brazil, Mexico, Peru, South Africa, Thailand, and the United States. After screening the eligible trial population for HIV infection to establish background incidence, participants who tested negative were randomized to receive injectable LEN every 6 months or daily oral TDF/FTC (*11*). The trial’s primary efficacy analysis compared HIV infection incidence in the LEN group with background HIV incidence in the screened population. The secondary efficacy analysis compared HIV infection incidence in the LEN group with the TDF/FTC group.

In both trials, study inclusion was based on the presence of sexual risk factors for HIV infection, and the randomized design was used for the secondary endpoint (daily oral PrEP use comparator), whereas the background incidence design was used for the primary endpoint (no PrEP use comparator) (Table 1) (*10*,*11*). In both trials, follow-up visits occurred at 4, 8, and 13 weeks, as well as every 13 weeks thereafter. Testing at follow-up visits included blood-based rapid antigen and antibody (antigen/antibody) tests and central laboratory antigen/antibody tests. HIV RNA testing was available for participants who acquired HIV infection during the trial (*10*,*11*).

A preplanned interim analysis was conducted when 50% of the target population had been enrolled for ≥52 weeks. After the interim analysis, independent data monitoring committees terminated the blinded phase of both trials based on the prespecified efficacy criteria^§^ (*10*,*11*).

### PURPOSE 1 Clinical Efficacy Trial

The PURPOSE 1 trial included a total of 8,094 females who were screened and included in calculating the background HIV incidence; 5,338 females were enrolled in the modified intention-to-treat (mITT) analysis in a 2:1:1 design (2,134: LEN plus an oral placebo; 2,136: TAF/FTC plus an injection placebo; and 1,068: TDF/FTC plus an injection placebo) (*10*). No new HIV infections occurred among females receiving injectable LEN over 52 weeks of follow-up. Within this group, LEN was 100% efficacious compared with no use of PrEP (incidence rate ratio [IRR] = 0; 95% CI = 0–0.04) and with TDF/FTC (IRR = 0; 95% CI = 0–0.10) (*10*). In the postprimary analysis, two participants with incident HIV infection were identified in the LEN group. One of the incident infections occurred in a participant after discontinuation of LEN during a period in which drug concentrations decreased below the target levels necessary for protection against HIV. No HIV-1 capsid inhibitor mutations were detected (*9*). The second participant in the LEN group with an incident HIV infection had a low HIV viral load, and genotyping to detect LEN resistance-associated mutations was not possible (*9*).

### PURPOSE 2 Clinical Efficacy Trial

The PURPOSE 2 trial included a total of 4,634 participants (see Table 1 in the trial report for details on demographics [*11*]) who were screened and included in calculating the background HIV incidence; 3,265 participants were enrolled in the mITT analysis in a 2:1 design (2,179 LEN plus an oral placebo and 1,086 TDF/FTC plus an injection placebo) (*11*). In this study, LEN was 96% efficacious in preventing HIV infection compared with no PrEP use (IRR = 0.04; 95% CI = 0.01–0.18) and 89% efficacious compared with TDF/FTC (IRR = 0.11; 95% CI = 0.02–0.51) (*11*). Two HIV infections occurred in participants receiving LEN; in both cases, drug concentrations were within ranges expected to be protective, and the HIV-1 capsid mutation N74D was detected. One of the participants with an incident HIV infection had a viral load of 14,000 HIV copies/mL; HIV diagnostic assays were notable for negative rapid test results and positive laboratory-based antigen/antibody test results at diagnosis. The other participant had a viral load of 934,000 HIV copies/mL; both the rapid and the laboratory-based antigen/antibody test results were positive at diagnosis (*11*). In the postprimary analysis, one additional incident HIV infection was identified in the LEN group, and Q67H/K70R mutations in the capsid gene were detected (*9*).

### Safety and Adverse Events

Rates of any adverse events of any grade (excluding injection site reactions) and of grade 3 (severe), 4 (life-threatening), or 5 (fatal) adverse events were similar in all study groups in both trials (*10*,*11*). Deaths that occurred were determined to be unrelated to the study drug. Adverse events of grades 3–5 were more prevalent in the TDF/FTC group. Injection site reactions were the most commonly reported adverse event in all study groups in both trials. They occurred more often in the LEN group (PURPOSE 1 = 68.8%; PURPOSE 2 = 83.2%), were mainly grade 1 (mild) or grade 2 (moderate), and predominantly included pain, subcutaneous nodules, and induration. Symptom intensity and frequency decreased with subsequent injections. LEN discontinuations related to injection site reactions were infrequent (PURPOSE 1 = 0.2%; PURPOSE 2 = 1.2%) (*10*,*11*). In the postprimary analysis, nodules persisted for a median of 350 days (IQR = 182–470 days) in the PURPOSE 1 trial and 297 days (IQR = 176–423 days) in the PURPOSE 2 trial (*9*). The median diameter of the largest nodule per participant was 1.2 inches (3.0 cm) (IQR = 0.8–1.6 inches [2–4 cm]) (*11*).

Among 510 pregnancies that occurred among 487 women in PURPOSE 1, pregnancy-associated adverse events and pregnancy outcomes were comparable in the 184 participants receiving LEN and those receiving tenofovir-based PrEP, both consistent with background rates; no adverse events were reported among breastfed infants exposed to LEN (*10*). No HIV infections occurred among pregnant women who received LEN; five infections occurred in the tenofovir-based PrEP groups (*10*).

The overall certainty of evidence for the five outcomes assessed was high based on GRADE. LEN is highly efficacious (Table 1) and safe (Table 2) as PrEP. The most commonly reported adverse event was an injection site reaction, which led to few discontinuations.

## Clinical Recommendation for Use of Lenacapavir for HIV Prevention

Lenacapavir, administered as subcutaneous injections every 6 months, is recommended as an HIV PrEP option in persons weighing ≥77 lbs (≥35 kg) who would benefit from PrEP. This is a strong recommendation, based on high certainty of evidence.

## Clinical Guidance for Use of Lenacapavir for HIV Prevention

### Identification of Persons Eligible to Receive LEN

Clinicians should consider PrEP in persons whose behaviors and epidemiologic context place them at risk for HIV acquisition (*12*). These populations include but are not limited to those with identified high HIV incidence in the United States (including persons with diagnosed sexually transmitted infections, men who have sex with men, persons whose sexual partners have HIV infection without viral suppression, persons who engage in transactional sex, persons who are incarcerated, and persons who share needles). PrEP should be offered in addition to other evidence-based HIV prevention strategies and within a comprehensive primary care strategy (*12*).

Clinicians should educate all eligible persons about current available PrEP options, discuss product choice, provide follow-up and medication adherence support, reassess ongoing risk, and align PrEP care goals accordingly (*12*). LEN might offer a good option for persons who prefer not to take a daily oral pill. The National Clinician Consultation Center PrEPLine (855-448-7737) and the NationalHIVPrEPCurriculum can provide guidance for prescribing PrEP medications.

Assessing eligibility for LEN consists of 1) assessing HIV acquisition risk, 2) excluding those with acute HIV infection, and 3) identifying significant drug interactions or contraindications (Box) (SupplementaryTable) (*9*,*17*,*18*). Based on the limited available safety and efficacy data, LEN PrEP may be used in pregnant women or continued in women who become pregnant while receiving injections, considering the woman’s risk for HIV without PrEP, after provider-client shared decision-making.

BOX Clinical considerations* for injectable lenacapavir for HIV preexposure prophylaxis — United States, 2025

**Eligibility**

- **Weight ≥77 lbs (≥35 kg) and at risk for HIV acquisition**^†^
- **No signs or symptoms of acute HIV infection.** If acute HIV infection is suspected, appropriate clinical care is recommended, including HIV RNA testing. 
- **Negative blood-based HIV antigen/antibody test result within 7 days before first lenacapavir (LEN) dose**
- **Negative confirmatory HIV RNA test, if available.** However, results of RNA assay should not delay initial LEN injection. Obtain up-to-date individual contact information to relay any positive test results and next steps.
- **Negative blood-based HIV antigen/antibody test result (no confirmatory RNA test needed) when switching from oral tenofovir-based or cabotegravir preexposure prophylaxis (PrEP) without interruption** ^§^
- **No known significant drug interactions or contraindications.** Significant drug interactions and supplemental LEN dosing are available from the Food and Drug Administration (FDA)in the LENprescribinginformation.

**Dosage**

Significant drug interactions and supplemental LEN dosing are available from FDA in the LENprescribinginformation.

- **Loading**
  - Day 1: 927 mg (3 mL) LEN by subcutaneous injection (two 1.5-mL injections at least 4 inches apart) and 600 mg orally (two 300-mg tablets)^¶^
  - Day 2: 600 mg LEN orally (two 300-mg tablets)
- **Maintenance:** Every 6 months (26 weeks) ±2 weeks, from the date of the last injection, 927 mg (3 mL) LEN by subcutaneous injection (two 1.5-mL injections at least 4 inches apart)^¶^

**Drug-Drug Interactions**

Significant drug interactions and supplemental LEN dosing are available from FDA in the LENprescribinginformation.

- Coadministration of strong and moderate cytochrome P450 3A (CYP3A) inducers requires supplemental LEN dosing.
- LEN increases the concentration of certain medications for up to 9 months after the last injection, requiring dose adjustments and monitoring for side effects.

**Initiation: First Visit**

- Assess eligibility and provide loading dose after shared decision-making.
- Provide information on adverse reactions, including injection site reactions, and recommendations, should a reaction occur.
- Review the schedule for follow-up visits and scenarios if an injection is missed.
- Screen for sexually transmitted infections (STIs) (vaginal, oral, rectal, urine, or blood, as needed) and viral hepatitis, and consider doxycycline postexposure prophylaxis.** Plan for follow-up STI screening based on risk, which might be more frequent than LEN visits.
- Provide support for behavioral risk reduction, follow-up, primary care, reproductive health, substance use, mental health, social needs, or other services, as needed.
- When switching clients from tenofovir-based PrEP, ensure they do not have hepatitis B virus (HBV) infection or are HBV immune. Initiate an alternate HBV treatment regimen for HBV-infected clients if tenofovir is discontinued. Provide HBV vaccination to clients who are not HBV immune.^††^

**Follow Up: Every 6 Months, or More Frequently as Needed**

More frequent interactions (e.g., texting and telephone calls) to follow up about adverse events, social support, and other care needs might be necessary, especially at the beginning of therapy; consider an initial follow-up at 1 month.

- Administer maintenance LEN injection.
- Assess for signs and symptoms of acute HIV infection. If acute HIV infection is suspected, appropriate clinical care is recommended, including HIV RNA testing.
- Negative HIV antigen/antibody test result.^§^
- Identify significant drug interactions or contraindications. Significant drug interactions and supplemental LEN dosing are available from FDA in the LENprescribinginformation.
- Assess for adverse reactions, including injection site reactions.
- Reassess HIV risk and PrEP needs and preferences.
- Screen for STIs (vaginal, oral, rectal, urine, or blood, as needed); consider doxycycline PEP.**
- Provide support for behavioral risk reduction, follow-up primary care, reproductive health, substance use, mental health, social needs, or other services as needed.

**Discontinuation of LEN**

- Inform PrEP users that when LEN is discontinued, medication levels in the body decline over 18 months, starting 6 months after the last injection, and do not offer HIV protection after 6 months; the period of declining medication levels over time is called the tail.
- Assess ongoing HIV risk and prevention plans and offer other PrEP options.

**Planned Missed Injection**

**Oral bridging (weekly oral maintenance) if a maintenance injection is anticipated to be ≥14 days late**

- Prescribe a maintenance dose of 300-mg LEN orally every 7 days for <6 months (<26 weeks). The first oral dose should be taken 6 months after the last LEN injection. Resume the maintenance LEN injection within 7 days after the last oral LEN dose.
- If oral LEN is not feasible during the planned missed maintenance injections, prescribe another form of PrEP until maintenance LEN injections resume.
- If maintenance injections cannot resume after 6 months of oral LEN bridging, then prescribe another form of PrEP.

**Unplanned Missed Injection**

**Maintenance injection ≥14 days late and oral LEN bridging has not been taken in the interim**

- Evaluate the reason LEN was missed, determine whether restarting is appropriate, and reinitiate as discussed previously (i.e., eligibility assessment, testing with HIV antigen/antibody and RNA tests, and prescribing, including loading dose).
- If LEN is not continued, see the Discontinuation section for counseling and offering other forms of PrEP.

**Time to Protection**

- Limited data suggest protective levels of LEN are achieved 2 hours after the dose on day 2, if both days of oral loading are taken (Lenacapavirdosinginspecialsituations:tuberculosisandbeyond).
- If both days of oral loading are missed, time to protection is estimated to be 21–28 days.

**Pregnancy, Lactation, and Breastfeeding**

- Limited human data on the use of LEN during pregnancy, lactation, and breastfeeding suggest no increases in drug-associated risks for adverse pregnancy, birth, and infant outcomes when compared with daily tenofovir-based PrEP or background rates.
- LEN PrEP may be used in pregnant women or continued in women who become pregnant while receiving injections, considering the woman’s risk for HIV without PrEP, after provider-client shared decision-making.
- Women should be encouraged to notify their providers if they are planning to conceive, become pregnant, or breastfeed.
- Health care staff members are encouraged to submit information on pregnancies during PrEP use to the AntiretroviralPregnancyRegistryonline or by telephone: 800-258-4263.

* The following clinical considerations are based on the 2021CDCPrEPClinicalPracticeGuideline and are supplemented with data from the FoodandDrugAdministration(FDA)label and other references. The recommendation updates the 2021 guideline by including lenacapavir (LEN) as an option for preexposure prophylaxis (PrEP).

^†^ Clinicians should consider PrEP in persons whose behaviors and epidemiologic context place them at risk for HIV acquisition (CDC. Preexposure prophylaxis for the prevention of HIV infection in the United States—2021 update: a clinical practice guideline. Atlanta, GA: US Department of Health and Human Services, CDC; 2021. https://stacks.cdc.gov/view/cdc/112360). These populations include but are not limited to those with identified high HIV incidence in the United States (including persons with diagnosed sexually transmitted infections, men who have sex with men, persons whose sexual partners have HIV infection without viral suppression, persons who engage in transactional sex, persons who are incarcerated, and persons who share needles).

^§^ See Recommended Laboratory Testing in report text for HIV screening recommendations. Perform confirmatory and other testing and initiate treatment for persons who receive positive HIV test results. For treatment of HIV infections acquired after receiving LEN, consider integrase strand-transfer inhibitor–based therapy (U.S.DepartmentofHealthandHumanServices|GuidelinesfortheUseofAntiretroviralAgentsinAdultsandAdolescentswithHIV2024).

^¶^ Injection sites include the abdomen (at least 2 inches away from the navel) and anterior thigh. Additional sites that are being studied include the upper arm (**Sources:** Lat AK, Kim A, Zhang H, et al. 1542. Impact of subcutaneous administration sites on the clinical pharmacokinetics of lenacapavir, a long-acting HIV capsid inhibitor: does body site matter? Open Forum Infect Dis Vol. 10 (Suppl. 2):2023. https://doi.org/10.1093/ofid/ofad500.1377; Clinicaltrials.gov. StudyofLenacapavirandEmtricitabine/TenofovirDisoproxilFumarate(F/TDF)inPreventionofHIVinCisgenderWomenintheUnitedStates(HPTN102)(PURPOSE3)).

** **Sources:** *STI screening:* CDCSTITreatmentGuidelines. *Hepatitis B screening:* Schillie S, Vellozzi C, Reingold A, et al. Prevention of hepatitis B virus infection in the United States: recommendations of the Advisory Committee on Immunization Practices. MMWR Recomm Rep 2018;67(No. RR-1). http://dx.doi.org/10.15585/mmwr.rr6701a1. *Hepatitis C screening:* InfectiousDiseasesSocietyofAmerica.HCVGuidance:RecommendationsforTesting,Managing,andTreatingHepatitisC. *Doxycycline PEP guidance:* Bachmann LH, Barbee LA, Chan P, et al. CDC clinical guidelines on the use of doxycycline postexposure prophylaxis for bacterial sexually transmitted infection prevention, United States, 2024. MMWR Recomm Rep 2024;73(No. RR-2). https://dx.doi.org/10.15585/mmwr.rr7302a1.

^††^ **Sources:** *HBV vaccination:* Weng MK, Doshani M, Khan MA, et al. Universal hepatitis B vaccination in adults aged 19–59 years: updated recommendations of the Advisory Committee on Immunization Practices—United States, 2022. MMWR Morb Mortal Wkly Rep 2022;71:477–83. https://dx.doi.org/10.15585/mmwr.mm7113a1. *HBV treatment:* AmericanAssociationfortheStudyofLiverDiseases|ChronicHepatitisBPracticeGuideline.

### Recommended Laboratory Testing

To rule out recent HIV infection, laboratory blood-based HIV antigen/antibody testing is recommended on the day of the initial LEN injection and with follow-up injections; however, LEN may also be administered if testing occurred within the preceding 7 days (Box). At initiation of LEN PrEP, a confirmatory HIV RNA test should be obtained in addition to the antigen/antibody test, if available; however, the results of the RNA assay should not delay injection (*9*). If HIV RNA testing is not available, a repeat laboratory-based antigen/antibody test should be scheduled in 4 weeks to exclude an undetected baseline infection (*12*). For those who receive positive HIV test results, confirmatory and other testing should be performed and treatment initiated; for treatment of HIV infections that were acquired after receiving LEN, integrase strand-transfer inhibitor-based therapy should be considered (GuidelinesfortheUseofAntiretroviralAgentsinAdultsandAdolescentswithHIV2024). 

If clinicians administer follow-up LEN injections based on a negative rapid HIV antigen/antibody test result, a confirmatory laboratory-based antigen/antibody test should also be obtained. Oral HIV antibody tests should not be used because of their low sensitivity in detecting recent HIV infection (*12*). If initiating LEN as a switch from tenofovir-based or cabotegravir PrEP without any interruption, only a laboratory-based HIV antigen/antibody test is needed before the injection. Tenofovir is an antiviral that can be used to treat chronic hepatitis B infection. Therefore, clinicians switching persons from tenofovir-based PrEP to LEN PrEP should determine whether the client has HBV infection or is immune to HBV. For persons with HBV infection, an alternative HBV treatment regimen should be provided if tenofovir is discontinued. HBV vaccination should be administered if a person is not immune to HBV (*19*,*20*).

### LEN Initiation and Injection Site Reactions

LEN is initiated with two 300-mg oral tablets (total 600 mg) each on days 1 and 2 (loading doses), along with two 1.5-mL subcutaneous injections (total 927 mg in 3 mL) administered in immediate sequence at least 4 inches apart on day 1 (*9*). Injection sites include the abdomen and anterior thigh (*9*). LEN forms a drug depot that might result in a subcutaneous nodule. Nodules are the most frequently reported injection site reaction. Nodules can be several centimeters in size and can last for several months to more than a year (*9*,*11*). Other injection site reactions include pain and induration. Incorrect injection technique, such as inserting the needle at an angle of ≤45° (resulting in intradermal rather than subcutaneous injection), is thought to contribute to injection site reactions (*11*). The manufacturer recommends an injection angle of 90°. Application of ice packs and use of topical and oral analgesics before and after injection might alleviate some signs and symptoms related to injection site reactions.

## Conclusion and Future Research

LEN is a highly effective and safe PrEP option administered every 6 months, which provides the potential to improve PrEP adherence and thus enhance HIV prevention in the United States. Additional research is needed to understand LEN’s effectiveness in persons who inject drugs, its ability to inhibit early viral replication if a person receiving PrEP becomes infected, the potential for emergence of resistance, safety and efficacy over longer follow-up periods, acceptability, and optimal strategies for implementation in clinical and community settings (*21*,*22*).

## Footnotes

* 45 C.F.R. part 46, 21 C.F.R. part 56; 42 U.S.C. Sect. 241(d); 5 U.S.C. Sect. 552a; 44 U.S.C. Sect. 3501 et seq.

† PURPOSE 1 trial inclusion criteria: 1) females; 2) aged 16–25 years; 3) sexually active with male partners; 4) not using PrEP; and 5) unknown HIV status or no HIV test within the 3 months preceding study enrollment. PURPOSE 2 trial inclusion criteria: 1) aged ≥16 years; 2) predominantly males; 3) have condomless receptive anal sex with male partners; 4) unknown HIV status; and 5) no HIV test or PrEP use within the 3 months preceding study enrollment.

§ An interim efficacy analysis was conducted when 50% of participants had completed 52 weeks of the study or discontinued the study early. The efficacy endpoint was the rate of HIV infection per 100 person-years in the LEN group compared with the rate of HIV infection per 100 person-years in the oral tenofovir-based PrEP group. The PURPOSE 1 and PURPOSE 2 trials were stopped early because LEN was found to be superior to no use of PrEP and to the use of TDF/FTC. As a result, the interim efficacy analysis constituted the primary efficacy analysis.
